# Supplementary material for: Trip duration drives shift in travel network structure with implications for the predictability of spatial disease spread
Source: PLoS Comput Biol. 2021 Aug 10;17(8):e1009127. doi: 10.1371/journal.pcbi.1009127 (PMC8378725; doi:10.1371/journal.pcbi.1009127)
Supplement: S1 Fig — The number of observed routes (A) and the number of observed trips (B) for each of the 20 duration-restricted travel networks. (PDF) [file pcbi.1009127.s001.pdf]

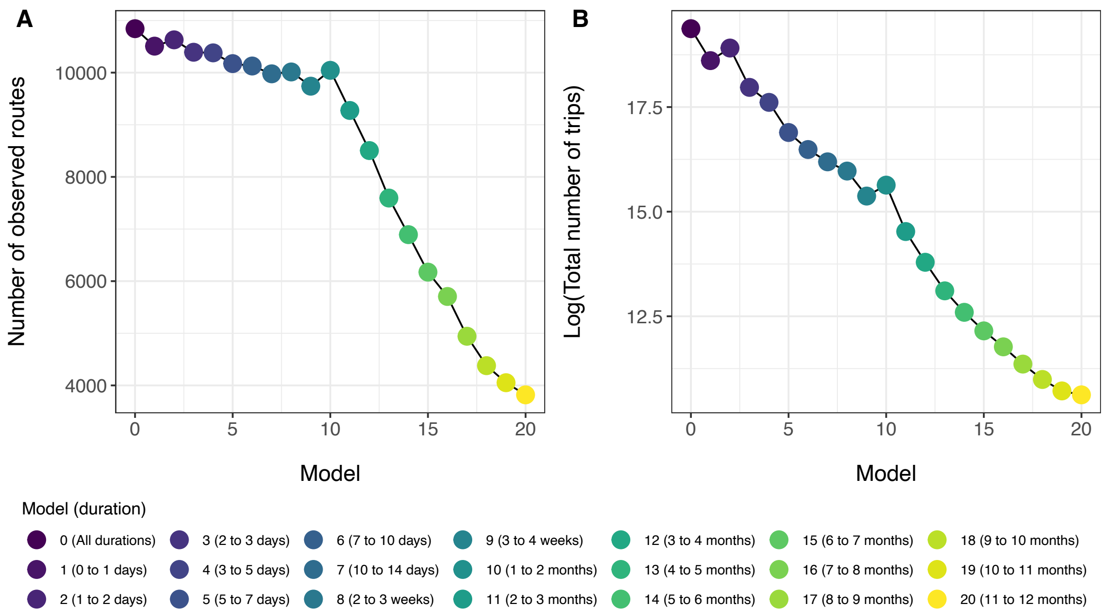

Figure S1: The number of observed routes (A) and the number of observed trips (B) for each of the 20 duration-restricted travel networks.
