# Supplementary material for: Trip duration drives shift in travel network structure with implications for the predictability of spatial disease spread
Source: PLoS Comput Biol. 2021 Aug 10;17(8):e1009127. doi: 10.1371/journal.pcbi.1009127 (PMC8378725; doi:10.1371/journal.pcbi.1009127)
Supplement: S3 Fig — Top: changes in the average expected value of for distributions of node strength ⟨r⟩ (A) and node closeness ⟨c⟩ (B) across all 20 of the duration-restricted travel networks. Bottom: changes in the variance of distribution of node strength ⟨r2⟩ (C) and node closeness ⟨c2⟩ (D) across all 20 of the duration-restricted travel networks. (PDF) [file pcbi.1009127.s003.pdf]

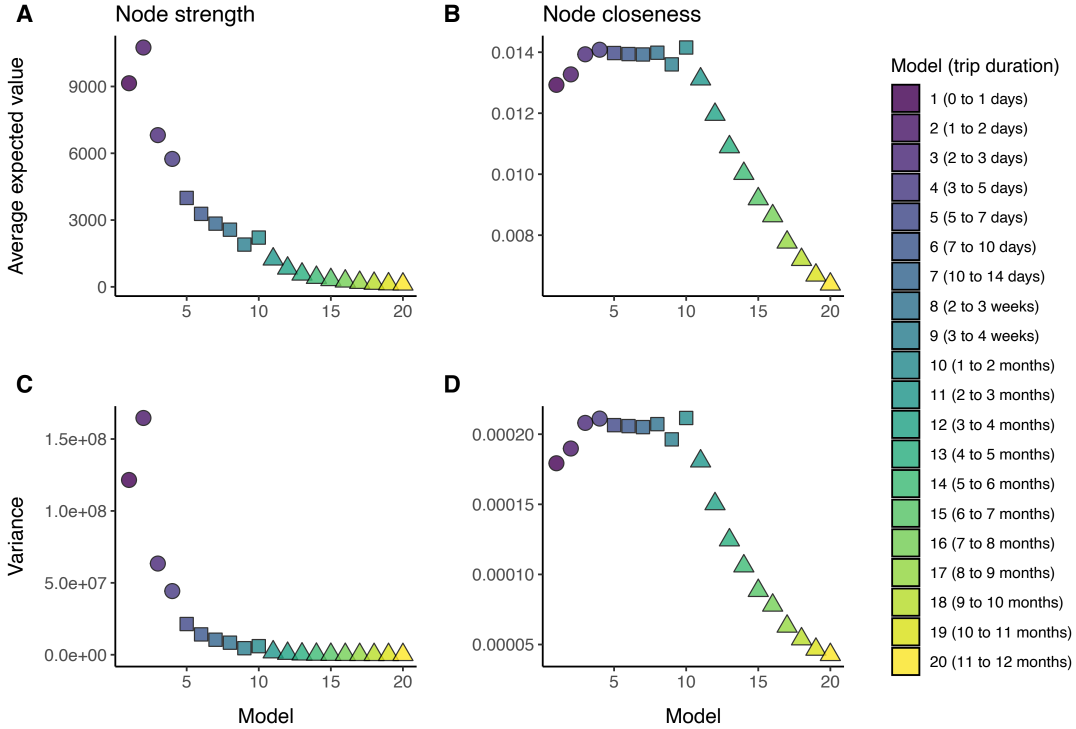


Figure S3: Top: changes in the average expected value of for distributions of node strength $\langle r \rangle$ (A) and node closeness $\langle c \rangle$ (B) across all 20 of the duration-restricted travel networks. Bottom: changes in the variance of distribution of node strength $\langle r^2 \rangle$ (C) and node closeness $\langle c^2 \rangle$ (D) across all 20 of the duration-restricted travel networks.
