# Supplementary material for: Trip duration drives shift in travel network structure with implications for the predictability of spatial disease spread
Source: PLoS Comput Biol. 2021 Aug 10;17(8):e1009127. doi: 10.1371/journal.pcbi.1009127 (PMC8378725; doi:10.1371/journal.pcbi.1009127)
Supplement: S4 Fig — Changes in node strength (A) and node closeness (B) for each duration-restricted travel network according to population density of the origin district. Points show the observed values of node strength and node closeness dependent upon the log population density of each origin district. Trend lines indicate the changes in both networks measures across all values of population density. The color key to the right indicates the duration interval corresponding to each travel network. (PDF) [file pcbi.1009127.s004.pdf]

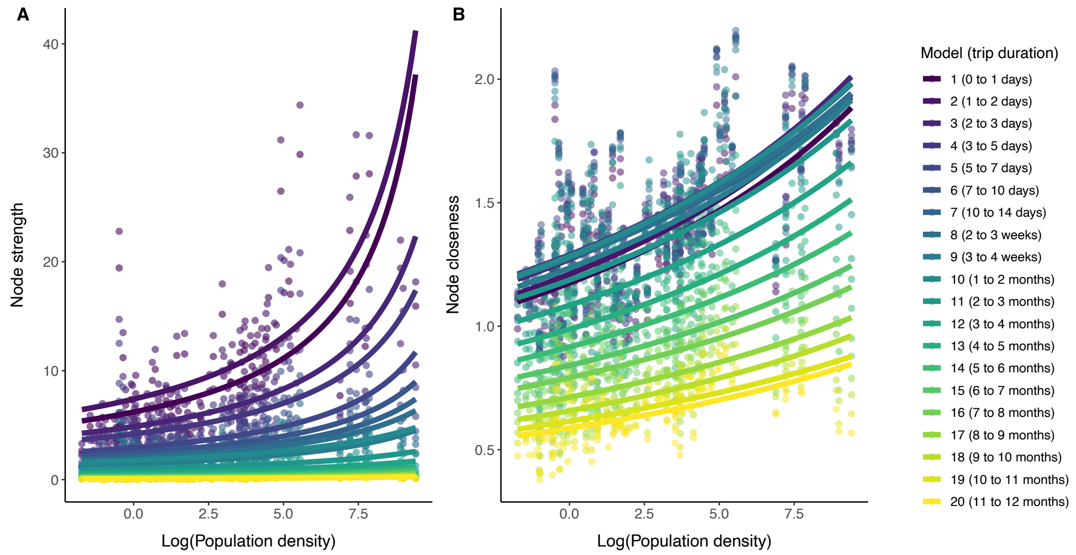

Figure S4: Changes in node strength (A) and node closeness (B) for each duration-restricted travel network according to population density of the origin district. Points show the observed values of node strength and node closeness dependent upon the log population density of each origin district. Trend lines indicate the changes in both networks measures across all values of population density. The color key to the right indicates the duration interval corresponding to each travel network.
