# Supplementary material for: Trip duration drives shift in travel network structure with implications for the predictability of spatial disease spread
Source: PLoS Comput Biol. 2021 Aug 10;17(8):e1009127. doi: 10.1371/journal.pcbi.1009127 (PMC8378725; doi:10.1371/journal.pcbi.1009127)
Supplement: S5 Fig — A) The relationship between the network heterogeneity metric based on node strength (ηr) and the average expected value of the observed distribution of node strength ⟨r⟩. B) The relationship between the network heterogeneity metric based on node closeness (ηc) and the average expected value of the observed distribution of node closeness ⟨c⟩. Dashed line indicates the x = y line and each point is colored according to duration-restricted travel network. (PDF) [file pcbi.1009127.s005.pdf]

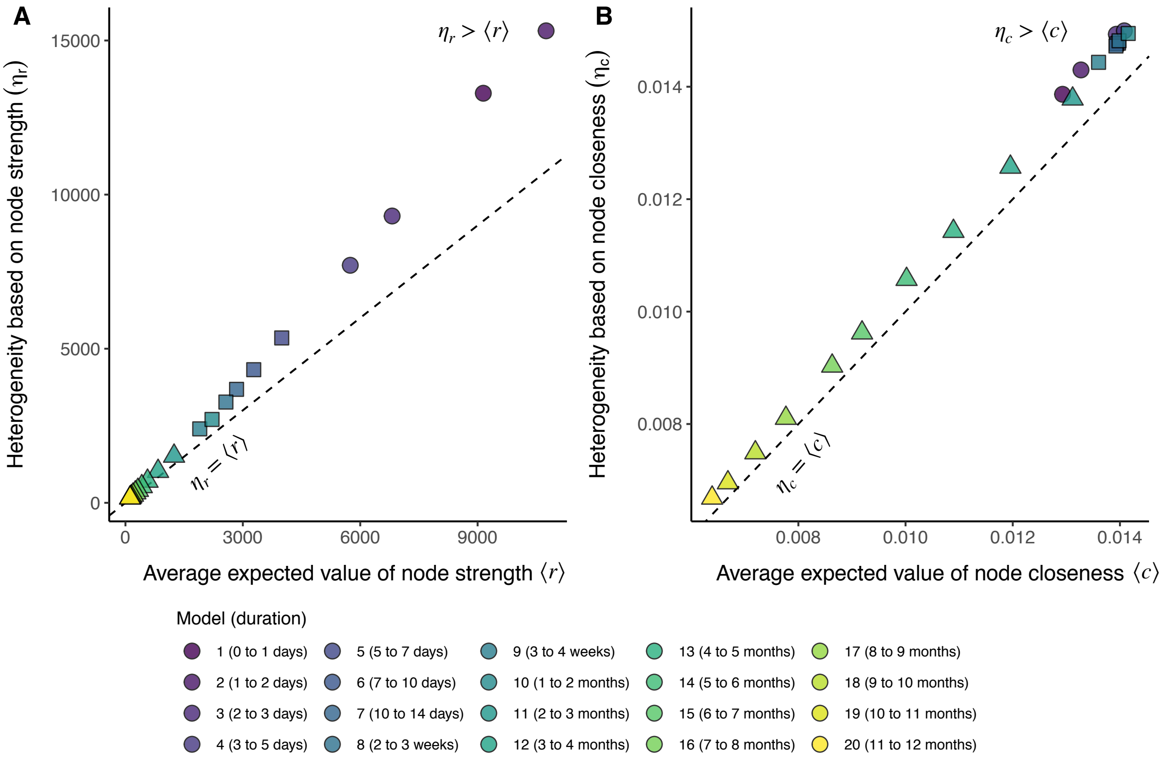

Figure S5: A) The relationship between the network heterogeneity metric based on node strength (η_r) and the average expected value of the observed distribution of node strength $\langle r \rangle$. B) The relationship between the network heterogeneity metric based on node closeness (η_c) and the average expected value of the observed distribution of node closeness $\langle c \rangle$. Dashed line indicates the $x = y$ line and each point is colored according to duration-restricted travel network.
