# Supplementary material for: Trip duration drives shift in travel network structure with implications for the predictability of spatial disease spread
Source: PLoS Comput Biol. 2021 Aug 10;17(8):e1009127. doi: 10.1371/journal.pcbi.1009127 (PMC8378725; doi:10.1371/journal.pcbi.1009127)
Supplement: S6 Fig — Figure A shows the R-squared value of each model and figure B shows the Mean Absolute Percent Error (MAPE). (PDF) [file pcbi.1009127.s006.pdf]

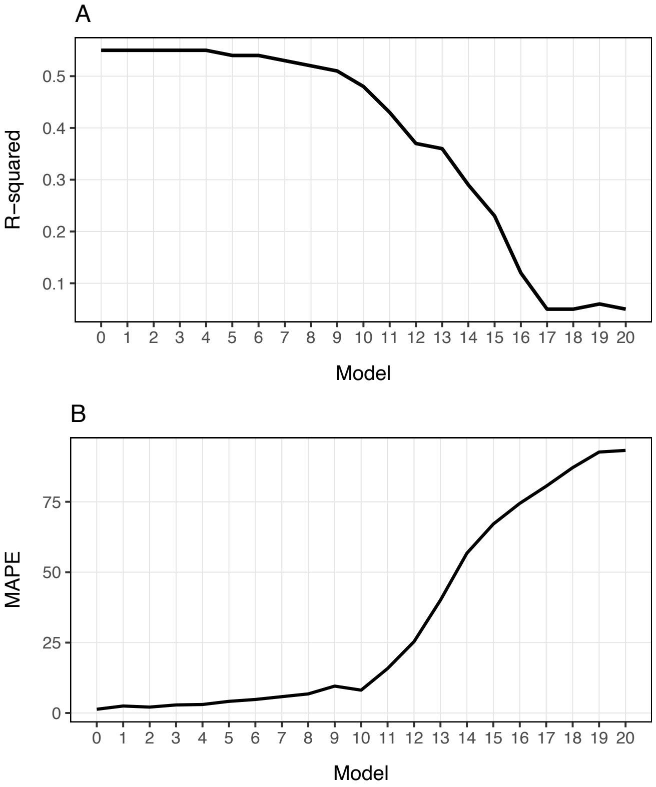

Figure S6: Change in goodness of fit across 20 duration-restrict gravity models. Figure A shows the R-squared value of each model and figure B shows the Mean Absolute Percent Error (MAPE).
