# Supplementary material for: Trip duration drives shift in travel network structure with implications for the predictability of spatial disease spread
Source: PLoS Comput Biol. 2021 Aug 10;17(8):e1009127. doi: 10.1371/journal.pcbi.1009127 (PMC8378725; doi:10.1371/journal.pcbi.1009127)
Supplement: S7 Fig — Down-sampled travel networks are the full travel network where the number of ob- served routes and number of observed trips is sampled randomly according to Table 1 in the main text. Figures A and C show changes in the distance parameter γ and population desti- nation size parameter ω2 respectively. Figure B shows the effective penalty of distance (dijγ) on estimated connectivity for each down-sampled network. Figure D shows the change in the attractive force of destination districts (Njω2) based on the population size of the destination for each of the down-sampled networks. (PDF) [file pcbi.1009127.s007.pdf]

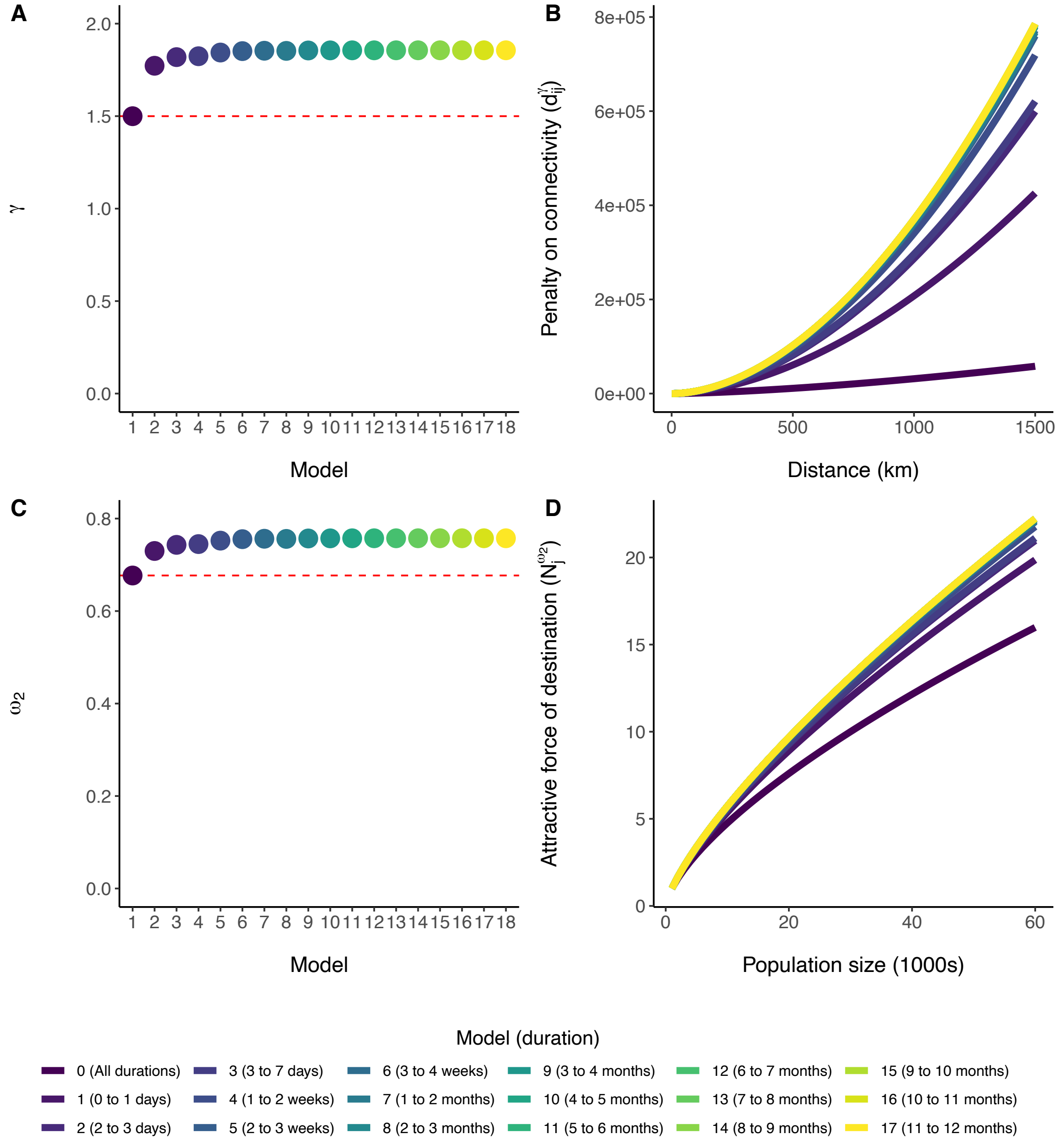

Figure S7: Changes in the gravity model parameters for each randomly down-sampled travel networks. Down-sampled travel networks are the full travel network where the number of observed routes and number of observed trips is sampled randomly according to Table 1 in the main text. Figures A and C show changes in the distance parameter γ and population destination size parameter ω_2 respectively. Figure B shows the effective penalty of distance (d_{ij}^{γ}) on estimated connectivity for each down-sampled network. Figure D shows the change in the attractive force of destination districts ($N_j^{\omega_2}$) based on the population size of the destination for each of the down-sampled networks.
