# Supplementary material for: Trip duration drives shift in travel network structure with implications for the predictability of spatial disease spread
Source: PLoS Comput Biol. 2021 Aug 10;17(8):e1009127. doi: 10.1371/journal.pcbi.1009127 (PMC8378725; doi:10.1371/journal.pcbi.1009127)
Supplement: S8 Fig — Down-sampled travel networks are the full travel network where the number of observed routes and number of observed trips is sampled randomly according to Table 1 in the main text. Red dashed line indicates the x = y relationship. (PDF) [file pcbi.1009127.s008.pdf]

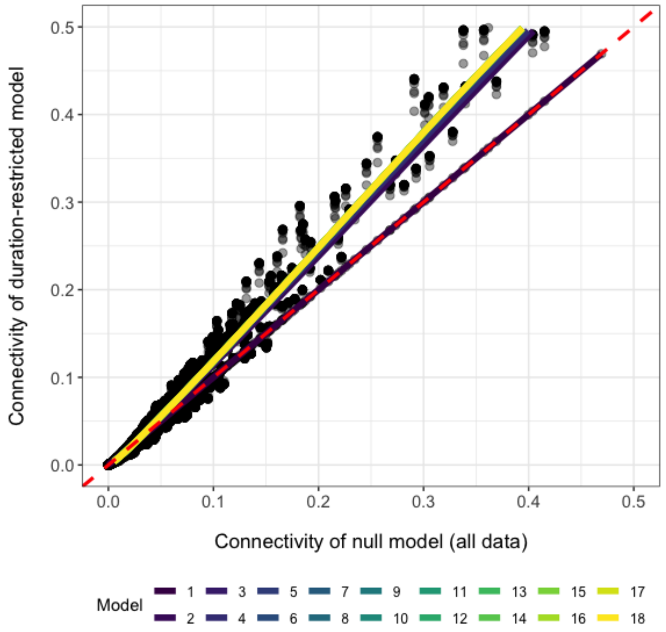

Figure S8: Connectivity values fitted by gravity models for all randomly down-sampled sub-models are compared to connectivity of the full model (all data). Down-sampled travel networks are the full travel network where the number of observed routes and number of observed trips is sampled randomly according to Table 1 in the main text. Red dashed line indicates the $x = y$ relationship.
