# Supplementary material for: Trip duration drives shift in travel network structure with implications for the predictability of spatial disease spread
Source: PLoS Comput Biol. 2021 Aug 10;17(8):e1009127. doi: 10.1371/journal.pcbi.1009127 (PMC8378725; doi:10.1371/journal.pcbi.1009127)
Supplement: S9 Fig — Covariates in the models include: distance between origin and destination, total population size (orig_pop, dest_pop), population density measured as people/km2 (orig_popdens, dest_popdens), and total administrative area (km2; orig_area, dest_area). Model fit was assessed with the Akaike Infor- mation Criterion (AIC = 2k − 2ln(L), where k is the number of parameters in the model and L is the estimated likelihood function). The circles represent the mean AIC for models across all origin location and all duration intervals with 50% and 95% confidence intervals shown as thick and thin lines respectively. The lowest AIC value of any model is shown by the dashed red line (AIC = 371) and the selected model (count ∼ distance + dest_pop) is indicated in light blue. (PDF) [file pcbi.1009127.s009.pdf]

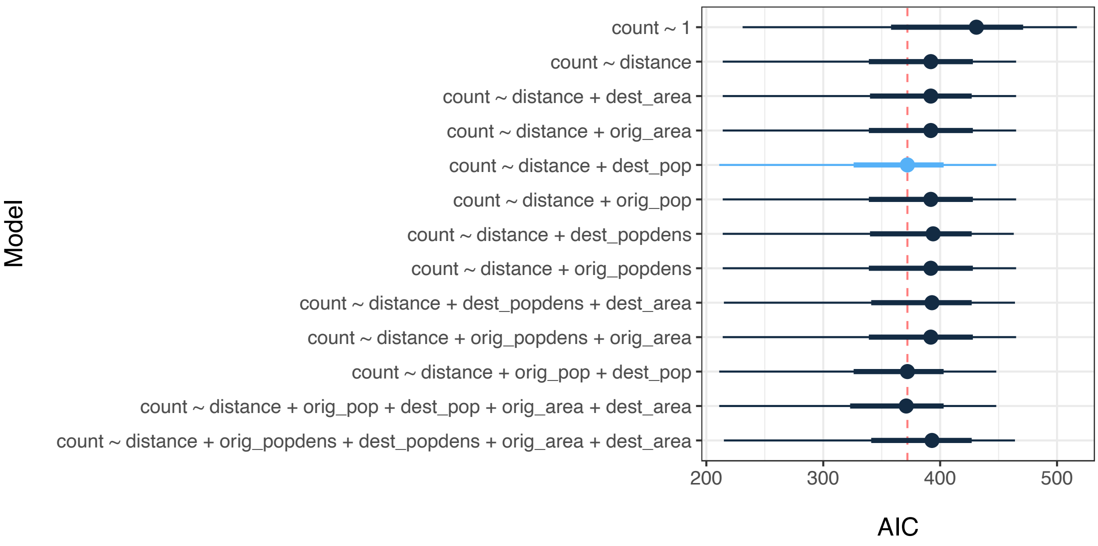

Figure S9: Model fitting results from 13 candidate log-linear models used to estimate trip counts among origin and destination locations within each of the 20 trip duration intervals. Covariates in the models include: distance between origin and destination, total population size (orig_pop, dest_pop), population density measured as people/km^2 (orig_popdens, dest_popdens), and total administrative area (km^2; orig_area, dest_area). Model fit was assessed with the Akaike Information Criterion (AIC $= 2k - 2\ln(L)$, where k is the number of parameters in the model and L is the estimated likelihood function). The circles represent the mean AIC for models across all origin location and all duration intervals with 50% and 95% confidence intervals shown as thick and thin lines respectively. The lowest AIC value of any model is shown by the dashed red line (AIC $= 371$) and the selected model (count $\sim$ distance $+$ dest_pop) is indicated in light blue.
