# Supplementary material for: Trip duration drives shift in travel network structure with implications for the predictability of spatial disease spread
Source: PLoS Comput Biol. 2021 Aug 10;17(8):e1009127. doi: 10.1371/journal.pcbi.1009127 (PMC8378725; doi:10.1371/journal.pcbi.1009127)
Supplement: S10 Fig — Example pathogens are indicated by colored lines: influenza, SARS-CoV-2, measles, Ebola, pertussis, P. falciparum malaria, and tuberculosis (TB). Colored lines represent the mean value of spatial predictability over 1000 stochastic TSIR simulations with shaded regions showing the 95% confidence interval for the mean. In these simulations, the capital district of Namibia, Windhoek East, was used as the introduction district. (PDF) [file pcbi.1009127.s010.pdf]

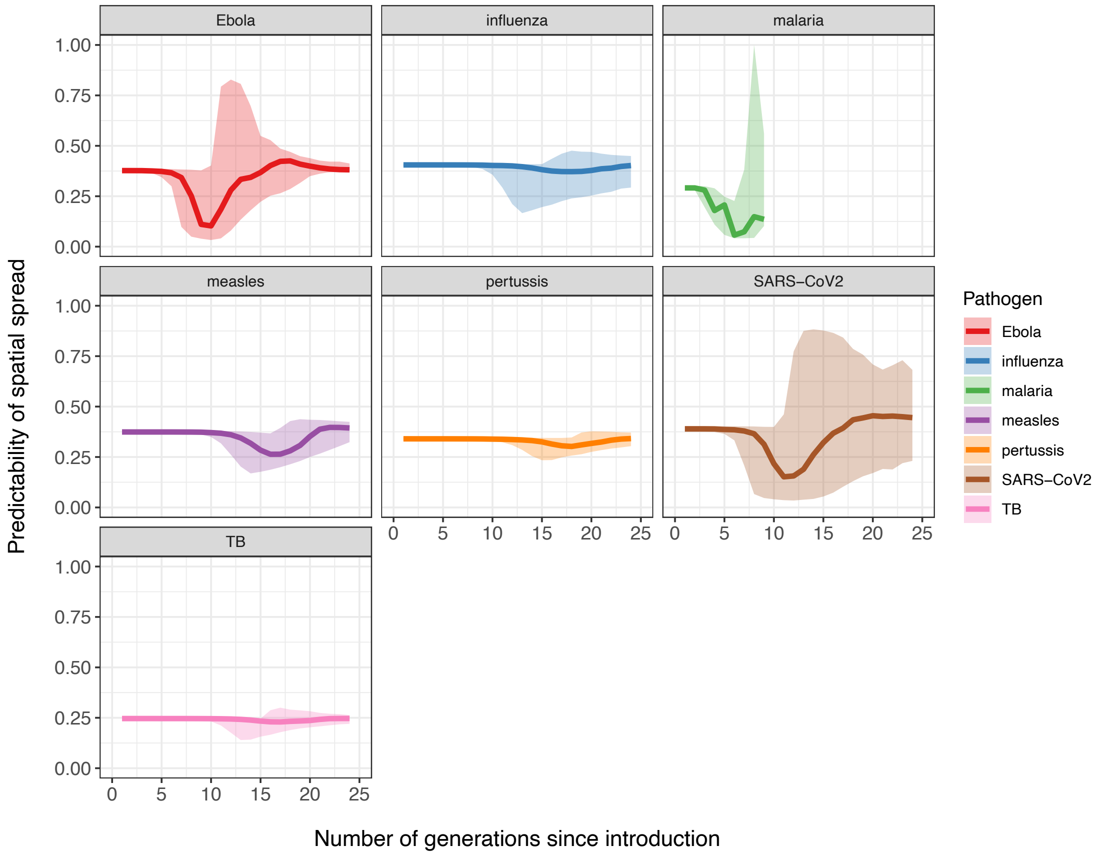

Figure S10: The change in spatial predictability over subsequent generations for 7 pathogens. Example pathogens are indicated by colored lines: influenza, SARS-CoV-2, measles, Ebola, pertussis, P. falciparum malaria, and tuberculosis (TB). Colored lines represent the mean value of spatial predictability over 1000 stochastic TSIR simulations with shaded regions showing the 95% confidence interval for the mean. In these simulations, the capital district of Namibia, Windhoek East, was used as the introduction district.
