# Supplementary material for: Trip duration drives shift in travel network structure with implications for the predictability of spatial disease spread
Source: PLoS Comput Biol. 2021 Aug 10;17(8):e1009127. doi: 10.1371/journal.pcbi.1009127 (PMC8378725; doi:10.1371/journal.pcbi.1009127)
Supplement: S11 Fig — The left figure shows the theoretical change in spatial predictability for a travel network with one infected origin location and 104 potential destinations (n = 105). The red circle indicates the maximum value of spatial predictability (φ = 0.56) based on spatial spread of several pathogens on the Namibia travel network. The figure on the right shows the change in spatial predictability for travel networks of up to n = 1000 locations. The travel network size is indicated by the color bar and the network with size equivalent to the Namibia travel network (n = 105) is highlighted in red. (PDF) [file pcbi.1009127.s011.pdf]

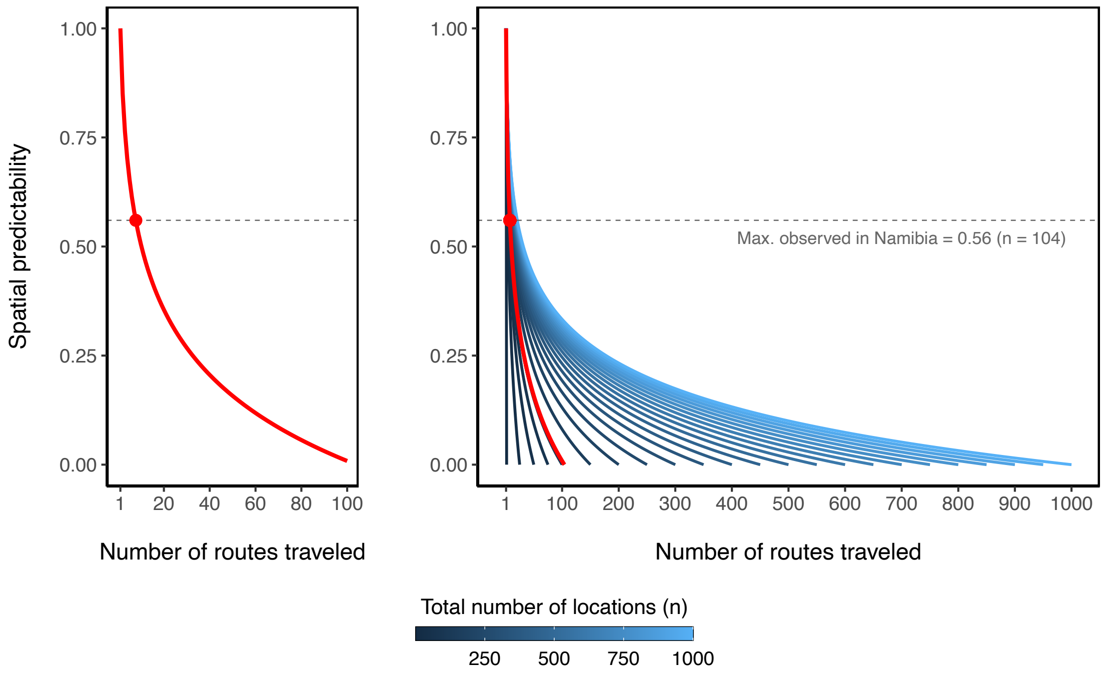

Figure S11: Change in spatial predictability depending on the number of destination locations that are predicted to have disease importation. The left figure shows the theoretical change in spatial predictability for a travel network with one infected origin location and 104 potential destinations ($n = 105$). The red circle indicates the maximum value of spatial predictability ($\phi = 0.56$) based on spatial spread of several pathogens on the Namibia travel network. The figure on the right shows the change in spatial predictability for travel networks of up to $n = 1000$ locations. The travel network size is indicated by the color bar and the network with size equivalent to the Namibia travel network ($n = 105$) is highlighted in red.
