# Supplementary material for: Trip duration drives shift in travel network structure with implications for the predictability of spatial disease spread
Source: PLoS Comput Biol. 2021 Aug 10;17(8):e1009127. doi: 10.1371/journal.pcbi.1009127 (PMC8378725; doi:10.1371/journal.pcbi.1009127)
Supplement: S1 Table — Where, R0 is the basic reproduction number, g is the generation time in days, s is the proportion of the population that is susceptible, and 1/γ is the infectious period in days. (PDF) [file pcbi.1009127.s012.pdf]

Table S1: Transmission parameters for each of the six pathogens used in simulations of spatial spread. Where, R_0 is the basic reproduction number, g is the generation time in days, s is the proportion of the population that is susceptible, and $1/\gamma$ is the infectious period in days.

Pathogen	R_0	g	s	$1/\gamma$	Citation
influenza	2 (1–3)	3 (2–6)	0.7 (0.6–0.8)	4 (3–5)	Boëlle et al. (2011)
SARS-CoV-2	3 (2–7)	8 (5–14)	0.95 (0.9–1)	7 (5.8–20)	Barber et al. (2020)
Ebola	1.5 (1–5)	14 (10–16.5)	0.95 (0.9–1)	6 (5–7)	Chowell and Nishiura (2014)
					Van Kerkhove et al. (2015)
measles	15 (12–18)	14 (12–16)	0.1 (0.05–0.3)	12 (10–14)	Anderson and May (1992)
pertussis	5.5 (5–9)	28 (25–33)	0.1 (0.05–0.15)	14 (12–16)	Vink et al. (2014)
					Beest et al. (2014)
malaria	10 (5–20)	60 (40–90)	0.75 (0.7–0.9)	20 (10–30)	Huber et al. (2016)
					Smith et al. (2007)
tuberculosis	4 (3–5)	180 (150–210)	1 (0.95–1)	60 (30–90)	Ma et al. (2018)
					Castillo-Chavez and Song (2004)

References

Anderson, R. M. and R. M. May (1992) *Infectious Diseases of Humans: Dynamics and Control*. OUP Oxford. 772 pp.

Barber, A., J. Griffin, M. Casey, Á. B. Collins, E. A. Lane, Q. T. Bosch, M. D. Jong, D. M. Evoy, A. W. Byrne, C. G. McAloon, F. Butler, K. Hunt, and S. J. More (2020) The basic reproduction number of SARS-CoV-2: a scoping review of available evidence. *medRxiv*, 2020.07.28.20163535.

Beest, D. E. te, D. Henderson, N. A. T. van der Maas, S. C. de Greeff, J. Wallinga, F. R. Mooi, and M. van Boven (June 1, 2014) Estimation of the serial interval of pertussis in Dutch households. *Epidemics*, **7**, 1–6.

Boëlle, P.-Y., S. Ansart, A. Cori, and A.-J. Valleron (2011) Transmission parameters of the A/H1N1 (2009) influenza virus pandemic: a review. *Influenza and Other Respiratory Viruses*, **5**, 306–316.

Castillo-Chavez, C. and B. Song (2004) Dynamical Models of Tuberculosis and Their Applications. *Mathematical Biosciences & Engineering*, **1**, 361.

Chowell, G. and H. Nishiura (Dec. 2014) Transmission dynamics and control of Ebola virus disease (EVD): a review. *BMC Medicine*, **12**, 196.

Huber, J. H., G. L. Johnston, B. Greenhouse, D. L. Smith, and T. A. Perkins (Sept. 22, 2016) Quantitative, model-based estimates of variability in the generation and serial intervals of Plasmodium falciparum malaria. *Malaria Journal*, **15**, 490.

Ma, Y., C. R. Horsburgh, L. F. White, and H. E. Jenkins (2018) Quantifying TB transmission: a systematic review of reproduction number and serial interval estimates for tuberculosis. *Epidemiology and infection*, **146**, 1478–1494.

Smith, D. L., F. E. McKenzie, R. W. Snow, and S. I. Hay (Feb. 20, 2007) Revisiting the Basic Reproductive Number for Malaria and Its Implications for Malaria Control. *PLOS Biology*, **5**, e42.

Van Kerkhove, M. D., A. I. Bento, H. L. Mills, N. M. Ferguson, and C. A. Donnelly (May 26, 2015) A review of epidemiological parameters from Ebola outbreaks to inform early public health decision-making. *Scientific Data*, **2**, 150019.

Vink, M. A., M. C. J. Bootsma, and J. Wallinga (Nov. 1, 2014) Serial Intervals of Respiratory Infectious Diseases: A Systematic Review and Analysis. *American Journal of Epidemiology*, **180**, 865–875.
