# Supplementary material for: Trip duration drives shift in travel network structure with implications for the predictability of spatial disease spread
Source: PLoS Comput Biol. 2021 Aug 10;17(8):e1009127. doi: 10.1371/journal.pcbi.1009127 (PMC8378725; doi:10.1371/journal.pcbi.1009127)
Supplement: Attachment — Submitted filename: Response_reviewers_20210330.pdf [file pcbi.1009127.s013.pdf]

To the editors-

We would like to thank the editor and reviewers for the helpful remarks and comments. In particular, we have clarified the structure and flow of our results to hopefully address comments by Reviewer 3. In addition, we have expanded our simulation model to include additional types of pathogens, more uncertainty and stochasticity in parameter estimates, and mobility models. Specific responses to each comment are provided below.

Author responses to reviewer's comments:

Reviewer #1: *This work provides an engaging analysis of the manner in which networks of human trips change based on the duration of the trip. It then develops potential implications of the more diffuse pattern of longer-duration travel for predicting the spread of an infectious disease from a point of introduction within the area. Although I have few criticisms of the network analysis, I have a hard time accepting the implications presented without further explanation about the assumed low-level details of how transmission occurs within the contact network.*

Response: Thank you for your thoughtful review of our manuscript. We agree that some of the simplifying assumptions could be improved to make our approaches more reflective of a wider range of transmission patterns. Therefore, we have revised both the manner in which the different trip duration networks are handled (allowing for a weighted combination based on trip duration) and have added more complex disease transmission dynamics as well. See responses below more detail.

The authors filter the full network of district-to-district trips extracted from CDR by trip duration, generating 20 duration-restricted subnetworks. In the simulation of disease spread, the authors consider pathogens with a range of reproduction numbers and generation times. For coupling populations, they use a gravity model that is fit to the duration-restricted subnetwork with a duration matched to the generation time of the pathogen. I don't understand why such a restricted contact model is appropriate, especially since the longer duration subnetworks comprise a much smaller volume of the total trips in the data sets. It would seem to me that trips of all duration are potentially relevant to the spread of any of the pathogens considered, and trips with a duration equal to the generation time of the pathogen may be relatively unimportant if they are sufficiently rare.

The rationale of the authors for using the duration-restricted networks seems to be primarily in the second paragraph of the discussion. The authors cite Keeling and Rohani (2002, Ecology Letters) as a theoretical precedent but I don't see a direction connection between the results in that paper, which analyze the dynamics around an endemic equilibrium, and the scenario modeled by the authors of this manuscript, which is movement away from an unstable disease-free equilibrium. Nevertheless, the authors write "[Keeling and Rohani] show that when the trip duration is shorter than the infectious period of the pathogen, interdependence between pathogen transmission parameters and coupling among locations increases." The authors may be referring in part to figure 2b of that paper, which shows that the coupling parameter increases with the return rate of the trip. That result seem to support my contention that the authors should not neglect trips that are of shorter duration than the generation time in their simulations.

Response: We agree with the points raised by the reviewer, in that a wider range of trips are relevant to transmission. While it is indeed true that we are dealing with early epidemic dynamics here rather than endemic dynamics, we were aiming to draw a parallel to previous work that has also noted the importance of scaling spatial connectivity based upon how long individuals stay in a destination. We also appreciate that this suggests shorter duration trips should be included. We have therefore updated the way the duration-restricted subnetworks are treated, such that each subnetwork now includes all trip durations, but they are each weighted relative to the generation time of the pathogen. Specifically, we define this as the weighted trip count: $(y_{ij} \mid g) = \sum_{t=1}^{T=g} y_{ijt} e^{-g/t}$, which decays based on the proportion of the generation time covered by the trip duration. Now, the total effective trip count is given by the sum of all weighted duration-restricted subnetworks up to a maximum duration that is equal to generation time. This allows trips of all durations to contribute to the structure of the travel network. In the case of shorter duration trips, the large number of these trips does have a significant impact, but this impact is progressively down-weighted as we consider longer generation times.

Keeling and Rohani (2002) also find that the proportion of time in a non-resident population is important for the extent of coupling. But this proportion depends not only on the duration of the trip (determined by the return rate tau in their model), but also on the frequency of the trip (determined by rho in their model). I point this detail out to put my comment in the previous paragraph about the rarity of long-duration trips making them potentially less important in the context of this theoretical work. Going back to the above quoted point in the manuscript, the authors next write "Accordingly, the spatial force of infection for pathogens with longer generation times is less skewed towards the distribution of shorter trips because the duration of shorter trips comprises a much smaller proportion of the total infectious period, allowing fewer opportunities for onward transmission." It seems from this sentence that I am not understanding exactly which results of Keeling and Rohani the authors have in mind, so I would recommend the authors being more explicit here. The authors are perhaps proposing some new theories in this and the following sentences based on the extent of overlap between trip duration and infectious period, which could very well be correct, but I find it difficult to understand them as written.

Response: We agree with the reviewer that our initial point was unclear. The particular finding in the Keeling and Rohani work that we wish to draw a parallel to is the relationship between proportion time-spent in non-resident population and the extent of spatial coupling. The authors report this when they state:

> *"although the movement patterns are determined by the population and are independent of the disease, the epidemiological differences in the infectious period mean that the precise value of [coupling] σ will be disease dependent. Analysis shows that it is the length of the infectious period relative to the average time spent away from home (τ/g) that determines the level of coupling"*

The reviewer is also correct that shorter duration trips are much more common than longer duration trips. However, this is balanced by the relationship between the frequency of these trips and the infectious period – for less common, longer duration trips with a long infectious period, these trips will contribute a substantial amount of infected person time. This is the point we aimed to make about fewer opportunities for onward transmission, that per trip there are fewer opportunities for short duration trips for pathogens with a longer infectious period. We have now updated the text accordingly:

"Similarly, we find that the spatial force of infection for pathogens with longer generation times is influenced by the distribution of shorter trips because the duration of shorter trips comprises a much smaller proportion of the total infectious period, allowing for less infected-person time in the visited location."

Minor comments:

1. The figures are generally well-constructed, but some figures (1, S3, S5) don't seem to have any references to them in the text. I just mention this in case it was an oversight of the authors.

Response: Thank you pointing these out- we have added these missing references to the text.

2. The y-axis on figure S4B would be more informative if it read "Node closeness".

Response: Thank you for the helpful suggestion we have now updated the figure.

3. The authors write that "pathogens with longer generation times may see more diffusive patterns of spread that are not dominated by urban travel." I wonder whether this apparent diffuseness is an artifact of the fact that there seems to be only a single urban center in the network. Perhaps if the data were available for the entirety of Southern Africa, one might see that longer duration trips were more affected by distance and population size. If the author see some merit to these ideas, I would suggest they caution against generalizing the results about the change in network structure with trip duration beyond the study area.

Response: This is an interesting point. It is true that Windhoek East and its surrounding districts are the major urban hub of Namibia, but there are also several other urban areas in the northern areas of the country (along the border with Angola) that provide population and geographic structure (see Figure 1B). While we do believe that our results would generalize to the other similarly rural, sparsely populated countries with a few population centers (common in many SSA countries), we are careful to not overstate this generality because it is plausible that different travel behavior will impact disease dynamics when looking at high-income countries or possibly at larger spatial scales. For example, the road networks and economic opportunities may be different in neighboring countries which may impact the underlying patterns.

4. Methods, Measures of network centrality, Is the a_ij in the equation for r_i a typo?

Response: No, the term in a_{ij} is the adjacency matrix (1 when node *i* and *j* are connected by an edge, 0 otherwise). This is taken from Barrat (2004, PNAS). We have updated the text with this detail.

5. Methods, Impact of trip duration and network structure on spatial disease dynamics, should

the equation for I_it = (beta s)^(t - 1) I would expect the susceptibility to affect growth in each generation.

Response: This is certainly true and in this first iteration we used a simple deterministic model with *s* as the overall population susceptibility, however we have updated the methods in response to the comments here and by Reviewer 3 so that transmission dynamics are modelled with a stochastic TSIR formulation that accounts for the depletion of the susceptible population.

<u>Reviewer #2:</u> *Giles et al present an exciting, novel study examining how large-scale travel patterns can drive the spread of different pathogens. Using a gravity model trained by Namibian cellphone data, they find that travel between districts is predictable using distance and population density, and they show that the duration of trips affects the predictability of the spread of different pathogens. I liked their approach, their figures are beautiful, and the conclusions could be important for tracking and predicting the spread of pathogens in the future. It was also well written, and whenever I thought I'd spotted a weakness or a desired clarification I read a few lines further and they had dealt with it, so I have relatively few comments. I commend the authors on a very thorough study. My one major comment is that it was unclear how they were treating their population size/density metrics, and I think it is important to make sure the metrics that were decided upon were 1) the best way to code it and 2) very precisely communicated. Upon resubmitting, I would recommend adding line numbers to make it a bit easier to attach specific recommendations.*

All the best,
Greg Albery

I was a bit confused about the measures of population size/density. Because the MS is in results-first format, I was under the impression that they were being fitted as densities (people per area) at some times and as sizes (absolute numbers of people, obtained by multiplying density by area) at others, but their reasoning was not entirely clear. Given that the input data were densities of cells and then they multiplied by area, I got a bit lost. This could maybe be solved by a table of terms, but that also could be overkill to solve this one density-specific problem. I'm happy to leave it to the authors, however they want to deal with it. A few elaborations:

Response: Thank you for bringing these details to our attention. We agree that there are subtle differences between using absolute population size and population density and have tried to clarify use of each throughout the text. In general, our reasoning is that absolute population size is the canonical measure used in gravity models, and in linear models of trip duration we found that a simple model with distance and destination population size was the best and most parsimonious. However, absolute population sizes are less intuitive when interpreting models due to the skewed population distribution in Namibia (Figure 1B). Therefore, we have used population density to in Figures 4 and 7 to add this context and indicate differences between urban and rural locations.

The waters were muddied a bit by the interchangeable use of size and density as terms in the MS, which contributes to a wider problem of size/density conflation in the literature. For

example, on page 7 "higher population" should be "denser"; in Figure 4B, the y axis says "density" and the caption says "size"; in Figure 7, the y axis includes the units "(people/km sq.)", which confused me further; on page 7, they refer to the "population size parameter ω2), which then made me think that density is used for the travel models, and sizes for the gravity models. This is understandable, I think, given the model specifications, and population density is a more universal measure than "population size of a specified area", but it is jarring to move between the two measures without clarification.

Response: This is a good point, we have changed the labels in Figure 4 to reflect the updated model, which uses total population sizes. Also, we have changed ambiguous wording such as 'higher population' to 'denser population' where appropriate.

Please can the authors be clearer throughout which measure precisely they are using, and check that they are using the two correctly? Did they ever try to fit cumulative population size (N) or geographic area (A) as an explanatory variable in the travel models, and/or were the travel patterns better described by density? Are these variables at all correlated?

Response: Yes, we have gone through the text an made sure to clarify when population size versus density is used. For the covariates in the models of trip duration, we have expanded the analysis of candidate models, which include now include covariates for the distance between origin and destination, as well as total population sum, population density (people/km^2), and total area of the administrative unit (km^2). Collinearity among these variables is negligible, as shown of the covariance matrix below.

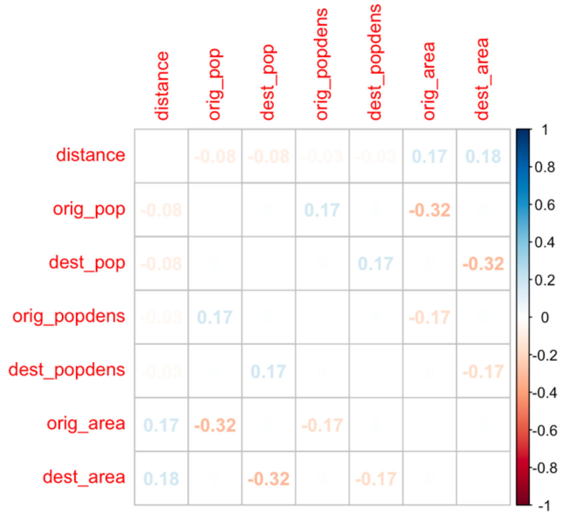

You could imagine a situation where larger (greater population size) districts attract more movement because they have more people and settlements, while greater density districts could be representative of important administrative centres or cities. There are also different implications offered by geographic area, which is doubtless correlated with population size.

Response: We agree. In fact, this is part of our reasoning for incorporating population density (in effort to bring in information on spatial area and distinguish between populations that are equivalent in size, but one is rural and spread out and the other is dense and developed). We

had not considered using area alone as a covariate however, so as mentioned above, we have explored this in these revisions. As one might expect, there is indeed a correlation between administrative area and total population size (-0.32), where the largest population sizes are most consistently associated with the smallest administrative areas. Although this correlation is appreciable, it is still low enough that we expect it not to impact model estimation.

If the authors did not attempt fitting these as explanatory variables I would recommend that they try, or justify why they chose density specifically and then be sure to explain how this relates to the population size metrics used in the gravity model. In an ideal world, I would also like to see population density, size, and geographic area (e.g. number of 100m grid cells) all compared, to ensure that their previous findings were not better described by the absolute population size or geographic area of the districts. If these are not overly correlated, then fitting multiple terms in the linear models could work, or a model selection procedure to see which best describes travel (using AIC).

Response: We appreciate this feedback on model testing and have incorporated this into the revised version of the manuscript. As mentioned above we have now included exploration of distance between origin and destination (km), as well as total population sum, population density (people/km^2), and total area of the administrative unit (km^2) as explanatory variables. We found that a model using distance and destination population size was sufficient to explain the variability in trip duration across all locations. The table below shows the comparison of the goodness of fit (measured by AIC) for 13 different model variants.

Model formula	Median AIC	Lower 95% CI	Upper 95% CI
count ~ distance + orig_popdens + dest_popdens + orig_area + dest_area	393	215	464
**count ~ distance + orig_pop + dest_pop + orig_area + dest_area**	**371**	**211**	**448**
**count ~ distance + orig_pop + dest_pop**	**372**	**211**	**448**
count ~ distance + orig_popdens + orig_area	392	214	465
count ~ distance + dest_popdens + dest_area	393	215	464
count ~ distance + orig_popdens	392	214	465
count ~ distance + dest_popdens	394	214	463
count ~ distance + orig_pop	392	214	465
**count ~ distance + dest_pop**	**372**	**211**	**448**
count ~ distance + orig_area	392	214	465
count ~ distance + dest_area	392	214	465
count ~ distance	392	214	465
count ~ 1	431	231	517

Here, the median AIC is measured across all locations with lower and upper 95% confidence intervals shown as well. While inclusion of administrative area did produce one of the lowest median AIC values, the confidence intervals for all models largely overlap. The three models in bold produced equally low median AIC values, therefore we selected the most parsimonious of the three as the best model (shown in green). Graphical representation of the above table has been added as Figure S9.

None of this affects the overarching message of the paper (which relied more on trip duration), nor could I see it doing so, but I think it's an important fundamental component of the paper that needs checking just to make sure the rest is on solid theoretical grounds (and for my own edification to make sure I understand what's going on).

Response: Indeed, we were happy to do a bit more formal model selection here and are glad to have the extra support for this section of the results.

Minor comments:

- *The abstract should mention the data that were used to derive the models.*

  Response: Done**.**

- *"time scale of transmission" needs defining when it first appears in the results.*

  Response: We have edited this sentence to read: "Changes in network topology are likely to impact the pattern of spatial spread for pathogens based on the time scale of transmission, which is determined by the generation time of the pathogen."

- *Generally, the "trip duration" aspect of the MS was well done, and very intriguing.*

  Response: Excellent to hear!

- *The discussion could remove the speculation about travel duration being restricted by cost. I can think of a number of reasons why these relationships might exist, depending on context, and I'm not sure the paper is helped by the point.*

  Response: We have now updated the discussion to be less speculative about costs – "These spatial differences in network topology may be driven by travel decisions stemming from the cost of travel, where the different classes of subnetworks may emerge from constraints on trips depending on the duration[64]. Accordingly, we find that short trips are more impacted by distance thus these trips concentrate around densely populated locations and show more heterogeneous structure like scale-free networks. But longer duration trips are less constrained by the cost of travel and therefore show more homogeneous network topology."

- *Figure 6 caption: "the" is repeated twice in the caption for B)*
    - *Needs a comma after Windhoek East*

  Response: Done**.**

<u>Reviewer #3:</u> Comments:
The study main's objective is to investigate the effects of human mobility on infectious diseases spread dynamics, with particular attention to trips duration and spatial heterogeneity. While this is a very interesting topic, and I appreciated the approach to subdivide the network according to trip duration, the manuscript is presented in a quite confusing flow and the results present little novelty.

Response: Thank you for your thorough review of our manuscript. We have taken into account your suggestions and made major revisions to the manuscript which we believe have improved the flow of ideas and communication of the novel aspects of this study. We have also made major revisions to the predictability analysis, where spatial dynamics are now modelled using a stochastic TSIR model that accounts for uncertainty in R0, generation time, and gravity model parameters. We no longer use networks with trip duration equal to the generation time, rather, spatial connectivity in the transmission models is a weighted combination of all trip duration networks.

The analysis on the trip duration defined networks is interesting from a network analysis perspective, although the choice of using 20 different sub-networks is unclear to me (se also comments below). It is also unclear why they imposed 3 classes of network, instead of estimating an optimal number of classes as well using the changepoint algorithm. This could be later connected with the epidemiology of diseases, providing a more fluid framework.

Response: The choice of 20 duration intervals was chosen to so that the intervals were comparable to the generation times of several notable pathogens and to show the changes in network structure as longer duration intervals are considered. We experimented with several different sets of duration intervals and this had no impact on the results. In terms of the classes of network, these were not chosen, but rather estimated by the changepoint algorithm as noted in line 694.

In my opinion, the predictability analysis needs to be reviewed. The variability embedded in most diseases' transmission dynamics is hardly considered, which makes it really hard to draw strong conclusions. First, it is unclear if the diseases spread has been calculated once in a deterministic way (using the model described in the methods) or over a number of simulations. Furthermore, All the parameters included (R0 and the generation time) are considered as points estimates, rather than distributions. As showed by the currently ongoing pandemic, the variance of the new cases generated by a single case can make a big difference, as well as the variability in generation time distributions. Here this aspect is not considered. This results in the analysis returning quite predictable results. For this reason, I believe that using single trips duration networks might be quite limiting, and (connecting with my previous point) would be more useful to consider an ensemble of sub-networks which could account for the generation time variability.

Response: We agree with the reviewer that our initial simulations were overly simplistic. We have now made major revisions to the disease transmission model. The model is now a fully stochastic TSIR model that allows R0 and generation time to vary according to ranges noted in Table S1. Uncertainty in the gravity model parameters is also accounted for. For each pathogen and point of introduction we performed 1000 replicates of the TSIR simulation model and

present the mean spatial predictability across all simulations. We have also revised the manner in which the travel networks are used in the disease simulations. Rather than have trip duration equal to generation time, spatial connectivity is now defined as the weighted combination of all trip duration networks so that each subnetwork now includes all trip durations, but they are each weighted based on the proportion of trip duration to generation time.

The choice of the diseases in the last analysis is odd. First, I would not consider malaria as a candidate, given its complex dynamics heavily affected by other environmental, climatic and ecological factors. These might indeed obscure the mobility and connectivity aspects.
Also, it is not clear why disease with slower dynamics are not considered (e.g. TB or HIV), since the data permit longer generation time analyses.
The manuscript would benefit from using empirical data of a real disease, to confirm the authors findings, but I understand if these are not available.

Response: The example pathogens that we have included were chosen based upon relevance to public health and to have various combinations of R0 and generation time. We agree that there are a number of additional drivers for malaria, but we do not claim that these relatively simple simulations are fully representative of each pathogen specifically. We included malaria as an example of a pathogen that has high R0 and a long generation time. We also appreciate the point about pathogens with much slower dynamics and have included a TB-like pathogen in the analysis.

Overall, the manuscript of a clear logic flow, it has been very challenging to understand all the analysis and their purpose in the study.

Response: Thank you for your detailed comments below- we have integrated these into this revision and believe that they have improved the clarity of the manuscript. These include light edits to the abstract to improve clarity and major revisions to the introduction that aim to improve the logical flow of ideas and introduction of specific terms. We have also added a fourth paragraph here that more clearly lines out the novel contributions of this study.

Minor comments
General
Even if it might not be specified by the journal guidelines, for next time please consider inserting line numbering and page numbers, and maybe use a 1.5 or 2 line spacing. These would quite help the reviewers.

Response: Done.

Abstract
• 2nd sentence: very confusing, please rewrite.
• 3rd sentence: see above.
• 4th sentence: “spatial distribution”
• 8th line: “imbedded”?
• Final sentence: please consider shortening.

Response: We have now updated the abstract to address these comments:
"Human travel is one of the primary drivers of infectious disease spread. Models of travel are often used that assume the amount of travel to a specific destination decreases as cost of travel increases with higher travel volumes to more populated destinations. Trip duration, the length of time spent in a destination, can also impact travel patterns. We investigated the spatial patterns of travel conditioned on trip duration and find distinct differences between short and long duration trips. In short-trip duration travel networks, trips are skewed towards urban destinations, compared with long-trip duration networks where travel is more evenly spread among locations. Using gravity models to inform connectivity patterns in simulations of disease transmission, we show that pathogens with shorter generation times exhibit initial patterns of spatial propagation that are more predictable among urban locations. Further longer generation time pathogens have more diffusive patterns of spatial spread reflecting more unpredictable disease dynamics."

Introduction
This section needs a strong revision. Sentences are often confusing and too long, and there is a lack of punctuation. From the point of view of content, it might also benefit from a re-organisation with a clearer flow and a better introduction of specific concepts. At the end of this section, the novelties presented by this study are not clear.

Response: We have done a major revision to the introduction and have specifically tried to make the sentences more concise with better flow between paragraphs. We have added brief elaborations to introduce specific concepts (e.g. return rate) and split the final paragraph with clearer statement of novelty.

1st paragraph
• *4th line: given that most of the COVID-19 pandemic happened in 2020 (so far), consider remove "2019" or add "started at the end of".*

Response: Done.

• *4th sentence: reference needed.*

Response: Done.

• *5th sentence: isn't this already stated at the beginning of the section? (1st sentence)*

Response: Agree, we have integrated this sentence into the one following it.

• *7th sentence: I would say that the challenges are caused by the complexity of the phenomenon we want to model, not because models "often exhibit complex dynamics ".*

Response: Agree, we have added mention of this.

3rd paragraph
• 2nd sentence: the part after the comma is not very clear, and please introduce "return rate" meaning.

Response: Agree, we revised this sentence and the one following to better introduce the meaning of return rate.

Results
General: many of the results are discussed in this section, and this creates an overlap with the discussion section. Please, consider presenting the results in a "cleaner" way, and then discuss them accordingly in the discussion section.

Response: We appreciate this perspective and have tried to clean up this section. We have removed several sections of text that are more appropriate for the discussion to improve the flow of this section and added minor revisions throughout to help clarify technical terms.

Figure 1 is never mentioned in the text, maybe it should go somewhere in the first Results paragraph.

Response: Done.

2nd paragraph
• 1st sentence: "we assessed travel by trip duration" please clarify.

Response: We have edited this to say: "To explore the impact of trip duration on connectivity patterns, we analyzed patterns of travel by assessing the spatial distribution of trips based on different trip durations."

• 3rd sentence: the phrase "duration-restricted subnetwork" is not very clear, maybe something like "subnetworks defined by trip duration" or similar would be more intuitive for the reader.

Response: Agree- done.

• 4th sentence: weighted by what?

Response: Node strength is defined as the node degree weighted by total number of trips. We have updated the text to clarify this point.

• 6th sentence: I am not sure of the meaning of this sentence, isn't the network clustering captured by the global clustering coefficient? Also please make clear that you calculated the heterogeneity according to Barrat's formula.

Response: Yes, we have removed this sentence and also added reference to the use of Barrat's formula.

• 6th to 9th sentences: this section would better fit the discussion. Also, a bit confusing and not entirely sure how useful is to discuss nodes strength or closeness "values" rather than their distribution.

Response: Agreed, we have removed these sentences to improve the flow of the results in this paragraph. And yes thank you for catching this, the use of 'values' here is in reference to the average expected value of the distribution. We have updated the text to reflect this.

• 10th sentence: by looking at figure S4 (and figure 3) I cannot see what the authors stated here. In particular, for short trip networks the closeness distribution looks quite "normaloid", rather than heavy tailed.

Response: This is a good point- we have changed the wording here so that the distributions for short trip networks are referred to as being flatter, rather than heavy-tailed, which implies skewness.

3rd paragraph
• 1st sentence: this would better fit the discussion. Also, please make clear where are the variances of strength and closeness showed.

Response: We have removed this sentence. Description of the distribution of node strength and closeness is covered in the preceding paragraph; however, we have edited the beginning of this paragraph so it is clear that we are talking about η_{strength} and η_{close} which do not have variances to speak of since they are summary statistics which describe the distributions of node strength and node closeness.

• Last two sentences: this would better fit the discussion.

Response: We have now moved these sentences.

4th paragraph
• Figure 4: this figure is very confusing, really hard to understand the message it tries to convey (if it is the findings described in this paragraph).

Response: We have now added a couple sentences to the beginning of this paragraph describing these regression models better. This now reads: "To understand factors driving differences in connectivity, we fit log-linear regression models to each of the 105 origin locations in the Namibia mobile phone data. For each location, the number of trips observed from the origin to each of its destinations was estimated using variables describing characteristics of the origin and destination: distance, total population size, population density (people/km2), and total administrative area (km^2). These models were applied to the trip counts within each duration-restricted subnetwork to understand how these covariates drive trip volume across progressively longer intervals of trip duration."

5th paragraph
• 1st sentence: “the most commonly used spatial interaction model”, citation needed. Moreover, the logic connection between the sentence and the table is not clear.

Response: Agreed, we have changed this to read: “We assessed the ability of a simple gravity model to capture the changes in spatial connectivity over each of the 20 duration-restricted subnetworks shown in Table 1.”

• Figure 5/A: is the reason that there are more than 1 value of connectivity for the full network because of subsampling? Please, clarify.

Response: No, the reason why there are multiple values for the full model in the Figure 5A is because we are plotting the individual connectivity values observed in the network. The connectivity matrix contains 105x105 elements. This figure shows the element-wise differences for fitted connectivity values of each of the duration-restricted models in comparison to the full model.

• R-squared ◊ R2

Response: Done.

6th paragraph
1st, 2nd and 3rd sentence: discussion.

Response: Done.

Discussion
1st paragraph
• 1st sentence: citation needed.

Response: Done.

2nd paragraph
• 3rd sentence: unclear, please rewrite.

Response: Done. Now reads: “They show that when trip duration is shorter than the infectious period of the pathogen, the effective level of spatial coupling among locations is reduced.”

• Last sentence: this is quite trivial.

Response: Agreed, we have removed this sentence.

3rd paragraph
• 1st sentence: citation needed.

Response: Done.

Methods
2nd paragraph: I am wondering which epidemiological difference it makes considering as separate networks built with 10 and 11 months (as a random example). Maybe longer period could be considered together (6-9 months, 9-12 months) to make the results easier to read, unless there is an epidemiological reason to keep them separate. This is also because no one of the considered disease has such long generation time, as well as the maximum assumed generation time is 30 days.

Response: We appreciate this suggestion since fewer intervals to refer to would indeed be easier to read but have chosen to keep the duration intervals as they are. This is mainly because the monthly intervals up to a year make a clearer demonstration of the changes in network structure over time which are shown in Figures 3—5.

4th paragraph: a bit confused, please rewrite. What is the purpose to use a gaussian kernel to estimate a statistical distribution, why the empirical could not be used? Moreover, it is not clear if at the end of this calculation we have 1 n_strength and 1 n_close value per each network, or a distribution of values.

Response: We have revised this paragraph to more clearly explain that η_{strength} and η_{close} are single metrics that summarize the statistical distributions of node strength and node closeness and that we calculate these specifically for the purpose of measuring the changes in network structure across networks defined by different intervals of trip duration. The use of a simple kernel density to estimate $P(r)$ was used because the empirical values of node strength and node closeness were sparsely distributed for some of the subnetworks. Calculating $P(r)$ this way is essentially the same as the empirical and has no impact on the presented results, it just makes the differences in heterogeneity across all of the subnetworks a little more continuous.

5th paragraph: I am not familiar with the specific algorithm used, but from what I read it looks like a clustering one. "Enforcing" is a quite odd wording choice.

Response: Agreed- we have changed this.

Last section: this needs a better explanation, it is really hard to follow and to understand the justification to the authors' choices. The connectivity parameter is calculated using a gravity model which is fitted to the observed data, correct? "Connectivity" is a vague term in this context, since in graph theory has other meanings (unless it has already been used with this precise formula, hence a reference would be necessary). Also, how does the transmission model accounts for the decline in susceptibility after the first generation?

Response: Thank you for flagging this- we have added this text to define connectivity in this context: “Connectivity values in are the estimated probabilities that a trip will go to each of the destinations when it departs origin, where each row in the matrix sums to 1.” The disease transmission model has undergone major revision and now accounts for the decline of the susceptible population using a stochastic TSIR model. The methods have been revised with a full description of the model.

Sup mat
Table S1: the reference for sars-cov-2 parameters is to a sars-cov-1 paper (2004). About the Ebola one, please considering to refer to more recent retrospective modelling studies rather than one published while the outbreak was still ongoing. Also, which influenza virus is considered here? The reference points to a book on disease modelling, quite vague.

Response: This is a good point. We have updated our references to use estimates from reviews and meta-analyses for SARS-CoV-2, Ebola, and influenza (H1N1). The citation for measles remains the Anderson and May book since transmission parameters for measles have not changed since these were published there.
